
JOURNAL INFORMATION
==============================
NLM Title Abbreviation: ScientificWorldJournal
Journal ID: ScientificWorldJournal
NLM Journal ID: 101131163
Journal ID: TSWJ

The Scientific World Journal

ISSN: 2356-6140
EISSN: 1537-744X
Publisher: Wiley

ARTICLE INFORMATION
==============================
PMCID: PMC6084730
PMID: 30147465
DOI: 10.1100/tsw.2001.152
Article ID: 694382
Article version: 1
Subjects: Short Report

Identification of Antiapoptotic Protein Kinases and Phosphatases by a Functional Genomics Approach

Abraham Reimar 1 *
Schubart Daniel 2
Ullrich Axel 1
1 Department of Molecular Biology, Max-Planck-Institut für Biochemie, Martinsried, Germany
2 Axxima Pharmaceuticals, Martinsried, Germany
*Reimar Abraham: abraham@biochem.mpg.de
Publication date: 2001
Electronic publication date: 2001 Oct 17
Volume: 1
First page: 32
Last page: 32
Copyright: Copyright © 2001 Reimar Abraham et al.
Copyright year: 2001
License: This is an open access article distributed under the Creative Commons Attribution License, which permits unrestricted use, distribution, and reproduction in any medium, provided the original work is properly cited.
License URL: https://creativecommons.org/licenses/by/3.0/

==============================
Miami *Nature Biotechnology* Short Reports
*TheScientificWorld* (2001) 1(S3), 32SR
ISSN 1532-2246; DOI 10.1100/tsw.2001.152

### IDENTIFICATION OF ANTIAPOPTOTIC PROTEIN KINASES AND PHOSPHATASES BY A FUNCTIONAL GENOMICS APPROACH

Reimar Abraham*, Daniel Schubart[+], and Axel Ullrich

Department of Molecular Biology, Max-Planck-Institut für Biochemie, Martinsried, Germany;
[+]Axxima Pharmaceuticals, Martinsried, Germany
abraham@biochem.mpg.de

**INTRODUCTION.** A lot of information has been gathered about the execution apparatus of apoptosis (1). But data on signals that control the initiation of apoptosis have only recently begun to be accumulated (2). In order to identify genes that regulate the process of apoptosis upstream of the apoptosis machinery we investigated the expression of signal transduction related molecules - mainly protein kinases and phosphatases - in Hela S3 cell lines that differ in their sensitivity towards apoptosis.

**METHODS.** Hela S3 cells were selected for survival after treatment with an apoptosis-inducing factor. Subsequently, the resistant cell clones were analyzed with respect to changes in their gene expression pattern.

**RESULTS.** The surviving clones were more resistant towards apoptosis than Hela S3. Analysis of the expression level of signal transduction related molecules revealed over 100 genes that are at least 2-fold upregulated in the clones when compared to Hela S3. Among the upregulated genes are well known antiapoptotic enzymes as well as enzymes that have not been linked to apoptosis before and need to be validated further.

Known antiapoptotic enzymes are for instance receptor tyrosine kinases like the EGF-receptor, HER-2 and the PDGF-receptor and tyrosine kinases like ryk. Inhibition of upregulated enzymes in the clones conferred an increased sensitivity to apoptosis proving that the upregulation indeed was responsible for the decreased apoptosis sensitivity.

Aside from known antiapoptotic proteins some proteins that are known to induce apoptosis are also upregulated suggesting that the interplay between apoptotic and antiapoptotic proteins determines the degree to which the cells are sensitive to apoptosis. These enzymes include for instance DAP-kinase, a serine threonine kinase that transmits apoptotic signals elicited by Fas (4) or csk, a tyrosine kinase that downregulates the activity of src-family kinases, which can act antiapoptotically.

Clustering of the expression data by a hierarchicalclustering method (3) found distinct clusters with proteins that could potentially upregulate and downregulate a common pathway. That corroborates the above notion of antiapoptotic and proapoptotic genes being regulated together.

**REFERENCES.**

1. Hengartner, M.O. (2000) Nature 407, 770-776
2. Rich, T., Allen, R.L., and Wyllie, A.H. (2000) Nature 407, 777-783
3. Eisen, M.B., Spellman, P.T., Brown, P.O., and Botstein, D. (1998) Proc. Natl. Acad. Sci. U S A 95, 14863-14868
4. Cohen, O., Inbal, B., Kissil, J.L., Raveh, T., Berissi, H., Spivak-Kroizaman, T., Feinstein, E. and Kimchi, A. (1999) J. Cell Biol. 146, 141-148
